# Diesel Aspiration Pneumonitis: A Rare but Serious Occupational Hazard

**DOI:** 10.7759/cureus.82977

**Published:** 2025-04-25

**Authors:** Hussain A Alwesaibi, Abdullah H Albin Saad, Mohammed S Almulaify, Majid G Alqatari

**Affiliations:** 1 Department of Internal Medicine, Dammam Medical Complex, Dammam, SAU; 2 Department of Pulmonary Medicine, Dammam Medical Complex, Dammam, SAU

**Keywords:** acute respiratory distress syndrome (ards), aspiration pneumonitis, chemical pneumonitis, diesel siphonage, hydrocarbon ingestion, hydrocarbon pneumonitis

## Abstract

Chemical pneumonitis is frequently encountered in patients following the siphoning of diesel fuel, which can lead to severe complications, including acute respiratory distress syndrome (ARDS).

This is a 33-year-old male patient who presented with a history of dyspnea, a non-productive cough, and heartburn. His symptoms commenced five days earlier following an inadvertent aspiration of diesel while refueling vehicles. This patient was admitted to our intensive care unit as a case of ARDS related to chemical pneumonitis following siphoning of diesel fuel. This patient was treated with intravenous steroids, along with empirical antibiotics, and intravenous furosemide was utilized for the management of non-cardiogenic pulmonary edema, as seen a few days post-admission and noted on chest radiography.

As the patient’s clinical condition improved, he was discharged after achieving a stable oxygen saturation of 97% on room air, accompanied by significant clinical recovery. Aspiration pneumonitis occurs following the inhalation of chemical irritants, including hydrocarbons, which can cause direct injury to the alveoli without significant systemic absorption, primarily through the disruption of surfactant function, leading to an inflammatory response and bronchial edema that can progress to ARDS.

This patient was then transferred to the regular ward after marked clinical improvement, where a CT scan of the chest was done, which shows bilateral segmental and sub-segmental consolidations with air bronchograms, along with peripheral patchy ground-glass opacities and atelectatic bands observed in the right middle lobe, lingula, and both lower lobes. Empirical antimicrobial therapy was initiated with intravenous piperacillin/tazobactam to provide coverage against potential anaerobic pathogens that may contribute to aspiration pneumonia. Systemic corticosteroids were also used as it has potential benefits in cases of ARDS.

Siphoning of diesel fuel can cause serious complications, including aspiration pneumonitis, which can end up with ARDS. Physicians should understand the pathophysiology by which diesel fuel can result in severe lung injury, aiming for early diagnosis and effective appropriate management.

## Introduction

Aspiration pneumonitis is a common complication encountered in individuals who siphon diesel fuel [1]. Chemical pneumonitis may arise from exposure to aerosols or the direct inhalation of liquid, and it can also occur secondary to aspiration during episodes of vomiting induced by diesel ingestion [2]. The siphoning of diesel leads to serious complications, including acute respiratory distress syndrome (ARDS), mucosal erosion of the oral cavity and esophagus, and pneumomediastinum [1].

## Case presentation

We report the case of a 33-year-old male patient from Nepal, with no significant medical history, who was brought to our emergency department with complaints of dyspnea, a non-productive cough, and heartburn. His symptoms began five days earlier following the accidental aspiration of diesel while refueling vehicles. The patient reported ingesting a small quantity of diesel and experiencing ocular exposure to the substance. He also complained of abdominal discomfort, denied any episodes of nausea or vomiting, and noted a reduced oral intake over the preceding days. There was no history of headache, dizziness, fever, skin rash, or other systemic symptoms.

On physical examination, the patient was awake, alert, and oriented to time, place, and person. He was in respiratory distress, with a respiratory rate of 42 breaths per minute. His general appearance was ill, with dry oral mucosa and bilateral conjunctival injection. Vital signs were as follows: blood pressure 170/92 mmHg, heart rate 82 beats per minute, temperature 37.2°C, and oxygen saturation of 92% on a simple face mask delivering oxygen at 7 L/min. Chest auscultation revealed bilateral coarse crackles across all lung fields. Abdominal examination was unremarkable, with a soft, non-tender abdomen. Cardiovascular examination revealed normal heart sounds, with no lower limb edema or signs of deep vein thrombosis.

Initial laboratory results showed leukocytosis, hypokalemia, and hyponatremia, along with conjugated hyperbilirubinemia, as shown in Table 1.

**Table 1:** Laboratory results.

Test	Result	Normal range	Units
White blood cells (WBC)	15	4.0-11.0	×10^9^/L
Hemoglobin	11.9	13.5-17.5	g/dL
Serum creatinine	98	62-115	µmol/L
Sodium (Na⁺)	128	135-145	mmol/L
Potassium (K⁺)	2.8	135-145	mmol/L
pH	7.4	7.35-7.45	-
Carbon monoxide	40	35-45	mmHg
Bicarbonate	27.3	22-28	mmol/L
Aspartate aminotransferase (AST)	88	10-40	IU/L
Alanine aminotransferase (ALT)	52	7-56	IU/L
Total bilirubin	56	3-21	µmol/L
Conjugated bilirubin	41	0-7	µmol/L
Gamma-glutamyl transferase (GGT)	164	8-61	IU/L
Alkaline phosphatase (ALP)	59	40-129	IU/L

**Figure 1:**
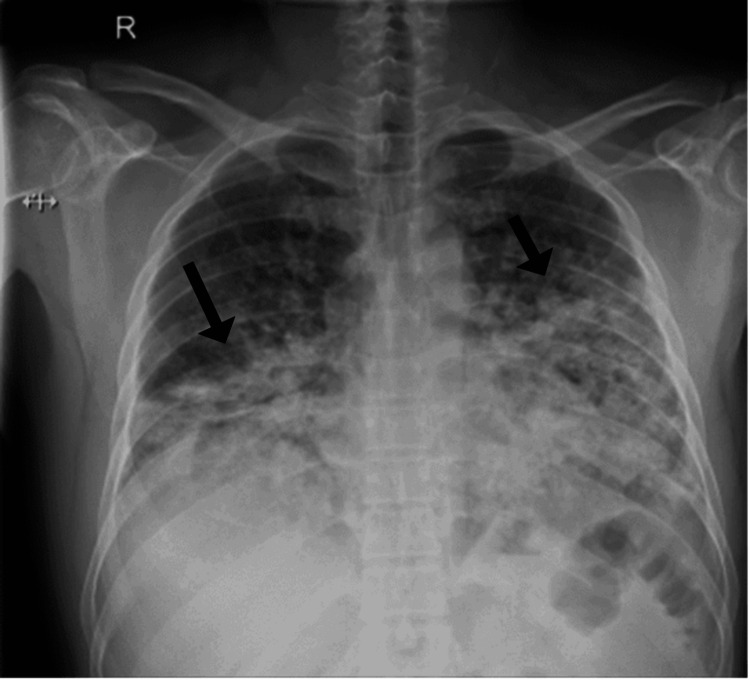
Chest radiography revealed bilateral heterogeneous opacities in the lower lung zones, suggestive of an airspace disease process (black arrows).

Chest radiography revealed bilateral heterogeneous opacities in the lower lung zones as shown in Figure 1.

The patient was admitted to the intensive care unit (ICU) with a diagnosis of aspiration pneumonitis complicated by ARDS, along with acute mixed-pattern liver injury attributed to the hepatotoxic effects of diesel exposure. Empirical intravenous piperacillin/tazobactam (4.5 g every six hours) and intravenous methylprednisolone (60 mg daily) were initiated. Electrolyte imbalances were corrected per ICU protocol. High-flow nasal cannula oxygen therapy was commenced (FiO_2_ 70%) due to persistent tachypnea, and intermittent non-invasive ventilation via bilevel positive airway pressure (BiPAP) was used to reduce respiratory effort. Chest physiotherapy, including incentive spirometry, was also employed to enhance airway clearance.

**Figure 2:**
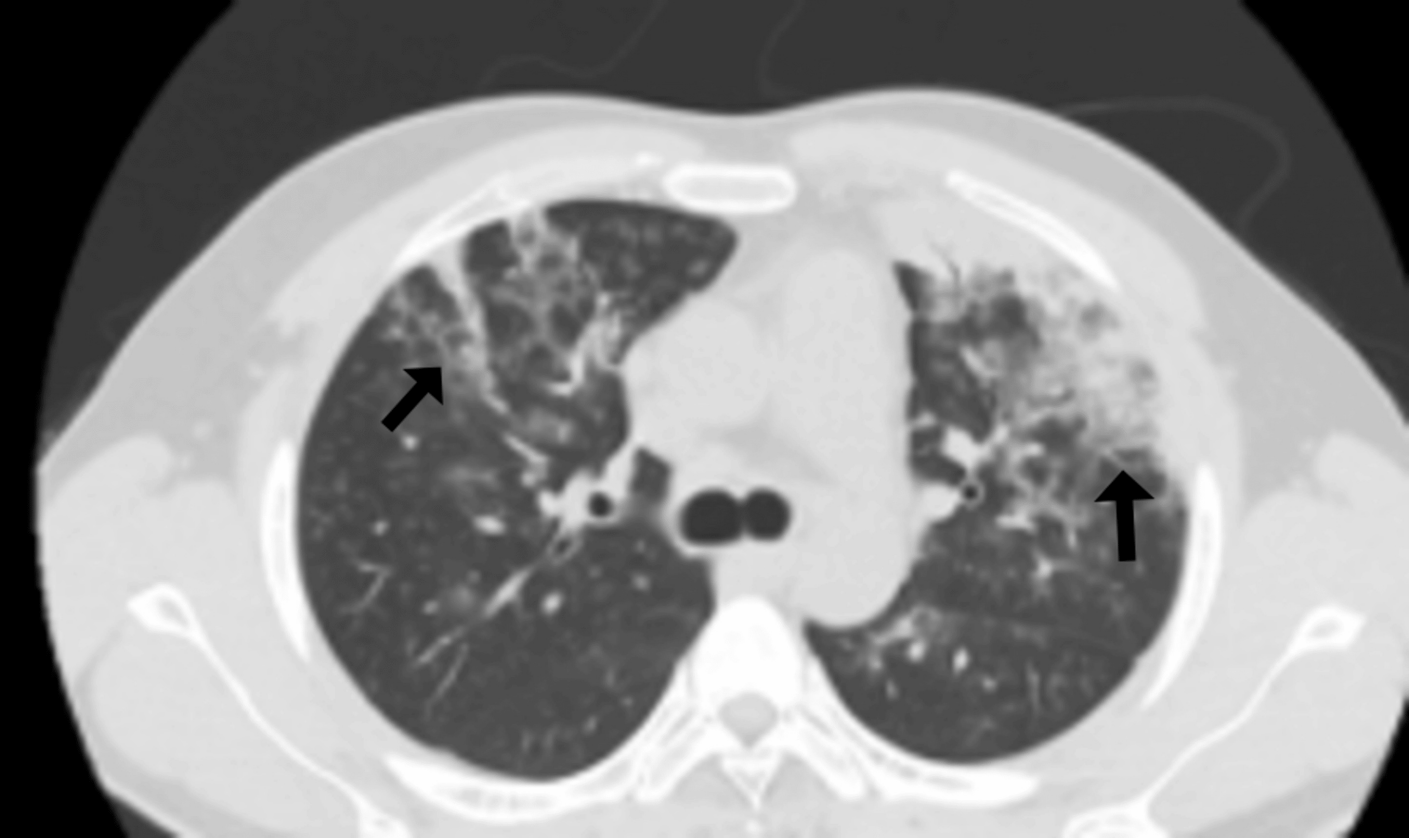
High-resolution computed tomography (HRCT) scan of the chest. Bilateral segmental and subsegmental consolidations with air bronchograms, along with peripheral patchy ground-glass opacities and atelectatic bands, were observed in the upper and middle lobes (black arrows).

**Figure 3:**
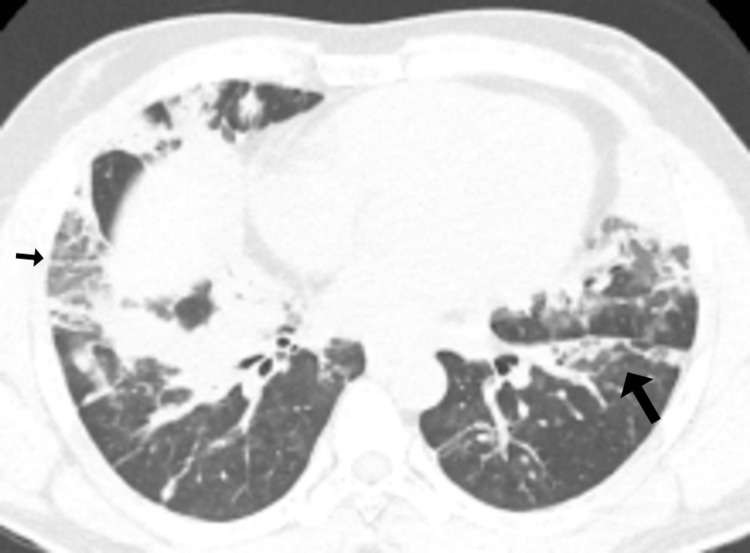
High-resolution computed tomography (HRCT) scan of the chest. Bilateral segmental and subsegmental consolidations with air bronchograms, along with peripheral patchy ground-glass opacities and atelectatic bands, were observed in the right middle lobe, lingula, and both lower lobes (black arrows).

Following clinical improvement, the patient was transferred to the general ward with an oxygen saturation of 96% on 4 L/min oxygen. Liver ultrasound showed a normal-sized liver (15.7 cm) with homogeneous echogenicity, no focal lesions, no intrahepatic ductal dilatation, and a patent portal vein (1.0 cm in caliber). The common bile duct measured 0.4 cm, and the gallbladder was unremarkable, with no evidence of cholelithiasis. Due to ongoing oxygen dependency, a high-resolution computed tomography (HRCT) scan of the chest was performed, which revealed bilateral segmental and subsegmental consolidations with air bronchograms, peripheral patchy ground-glass opacities, and atelectatic bands involving the right middle lobe, lingula, and both lower lobes as shown in Figures 2-3.

Blood cultures showed no microbial growth. Liver enzymes and bilirubin levels gradually normalized (total bilirubin decreased to 7 µmol/L). Hepatitis B surface antigen and hepatitis C serology were negative. The patient was discharged in stable condition, maintaining oxygen saturation of 97% on room air, with significant clinical recovery.

## Discussion

Aspiration pneumonitis occurs following the inhalation of chemical irritants, such as hydrocarbons. The underlying pathophysiology involves direct alveolar injury without significant systemic absorption. This injury is primarily mediated through surfactant disruption, resulting in an inflammatory cascade and bronchial edema, which may progress to ARDS. These pathological changes arise from an inflammatory response triggered by macrophage activation and the release of pro-inflammatory cytokines [1,2]. Differentiating between chemical pneumonitis and aspiration pneumonia is critical, as both conditions can present with overlapping symptoms such as cough, dyspnea, and, occasionally, fever [3].

In the present case, empirical antibiotic therapy with intravenous piperacillin/tazobactam was initiated to provide coverage against potential anaerobic organisms associated with aspiration pneumonia. While the role of corticosteroids in aspiration pneumonitis remains controversial, they have demonstrated potential benefit in cases of ARDS secondary to chemical injury [4]. Based on this rationale, intravenous methylprednisolone was administered.

Hepatic involvement following hydrocarbon aspiration has been previously documented in case reports and small studies [5]. In our patient, transient elevations in hepatic transaminases were observed: alanine aminotransferase (ALT) peaked at 527 IU/L before declining to 460 IU/L and subsequently 358 IU/L, while aspartate aminotransferase (AST) increased to 273 IU/L and later improved to 99 IU/L. These changes were attributed to a mixed-pattern liver injury secondary to diesel exposure.

Additionally, the patient developed non-cardiogenic pulmonary edema several days after admission, as evidenced by chest radiographic findings. Intravenous furosemide was administered to manage fluid overload and respiratory compromise associated with this complication.

## Conclusions

This case highlights the potential for significant pulmonary and hepatic complications following accidental diesel aspiration, an uncommon but clinically important scenario. The patient developed aspiration pneumonitis progressing to ARDS, alongside transient mixed-pattern liver injury, likely due to the hepatotoxic effects of hydrocarbon exposure. Prompt recognition and supportive management, including empirical antimicrobial therapy, corticosteroids for ARDS, oxygen supplementation via high-flow nasal cannula, and non-invasive ventilation, contributed to a favorable outcome.

The case underscores the importance of differentiating chemical pneumonitis from infectious aspiration pneumonia, as the clinical management and prognosis differ. Additionally, clinicians should remain vigilant for systemic effects, such as hepatotoxicity and non-cardiogenic pulmonary edema, in patients with hydrocarbon exposure. Timely supportive care, close monitoring, and a multidisciplinary approach remain essential in optimizing patient recovery and preventing complications.
